# Identification, Evaluation, and Correction of Supracrestal Tissue Attachment (Previously Biologic Width) Violation: A Case Presentation With Literature Review

**DOI:** 10.7759/cureus.58128

**Published:** 2024-04-12

**Authors:** Mohammad Nazish Alam, Wael Ibraheem, Karthikeyan Ramalingam, Sathya Sethuraman, Syed Nahid Basheer, Syed Wali Peeran

**Affiliations:** 1 Preventive Dental Sciences, College of Dentistry, Jazan University, Jazan, SAU; 2 Oral Pathology and Microbiology, Saveetha Dental College and Hospitals, Saveetha Institute of Medical and Technical Sciences, Saveetha University, Chennai, IND; 3 Physiology, Saveetha Dental College and Hospitals, Saveetha Institute of Medical and Technical Sciences, Saveetha University, Chennai, IND; 4 Restorative Dentistry, College of Dentistry, Jazan University, Jazan, SAU

**Keywords:** supra crestal fiberotomy, slow orthodontic extrusion, restorative margin, rapid orthodontic extrusion, precision, ferrule, dental crown, deep margin elevation, biological width, biological width violation

## Abstract

The supracrestal tissue attachment (SCTA) is the new terminology for biologic width. SCTA is defined as the physiologic dimension of a solitary functional unit composed of junctional epithelium and connective tissue attachment. Its preservation is critical for the well-being of periodontal health. SCTA has been widely studied and scientific literature is indicative of its significance during the placement of restoration, including prosthetic crowns. This should be taken care of in cases of anterior teeth within the smile zone, where dental crowns are regularly placed subgingivally for aesthetic reasons. In addition, any violation of SCTA while restoring the dentition will present as gingival inflammation and pain, consequently, leading to failure of the clinical procedure.

## Introduction

Maintenance of supracrestal tissue attachment (SCTA), previously referred to as biologic width, is crucial for periodontal health. This attachment comprises the junctional epithelium and the supracrestal connective tissue surrounding each tooth [1]. The concept of SCTA being vital for gingival health and, concomitantly, the success of restoration is well known and incorporated in clinical practice. The SCTA acts as a barrier and prevents the penetration of microorganisms into the periodontium [2]. Compromising this attachment can result in inflammation of the marginal gingiva, accelerated bone loss, and increased pocket depth [3,4]. Common causes of SCTA violation include incorrectly placed restoration margins, leading to chronic inflammation [5,6].

Preserving the proper dimensions of the SCTA is vital for overall periodontal health. The ideal SCTA width consists of 1 mm of supracrestal connective tissue attachment, 1 mm of junctional epithelium, and 1 mm for sulcus depth [7]. Deviation from this SCTA width can cause attachment loss and pocket formation, underscoring the importance of preventing such violations during dental procedures [5]. There are three restorative margins for prosthesis placement: supragingival, equigingival, and subgingival. The concern about SCTA violations arises from the deep placement of subgingival margins, primarily used to achieve better aesthetics.

We present a case related to SCTA and a relevant clinical application review in this manuscript.

## Case presentation

**Figure 1:**
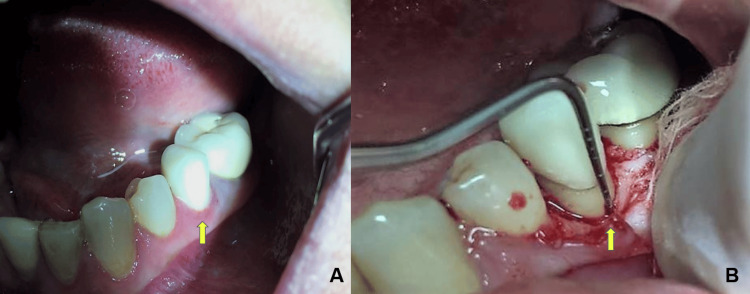
Clinical images A: Intra-oral picture showing gingival inflammation in teeth 35 region B: Intra-operative picture showing periodontal measurement during crown lengthening with apically displaced flap and osteotomy Image credit: Mohammad Nazish Alam

A 47-year-old female patient reported to clinics complaining of discomfort concerning teeth 35 and 36. The concerned teeth had prosthetic crowns which were placed a few weeks earlier. The patient complained of discomfort post-placement of the crown with teeth 35. Clinical examination revealed inflammation and bleeding on probing (Figure 1). 

**Figure 2:**
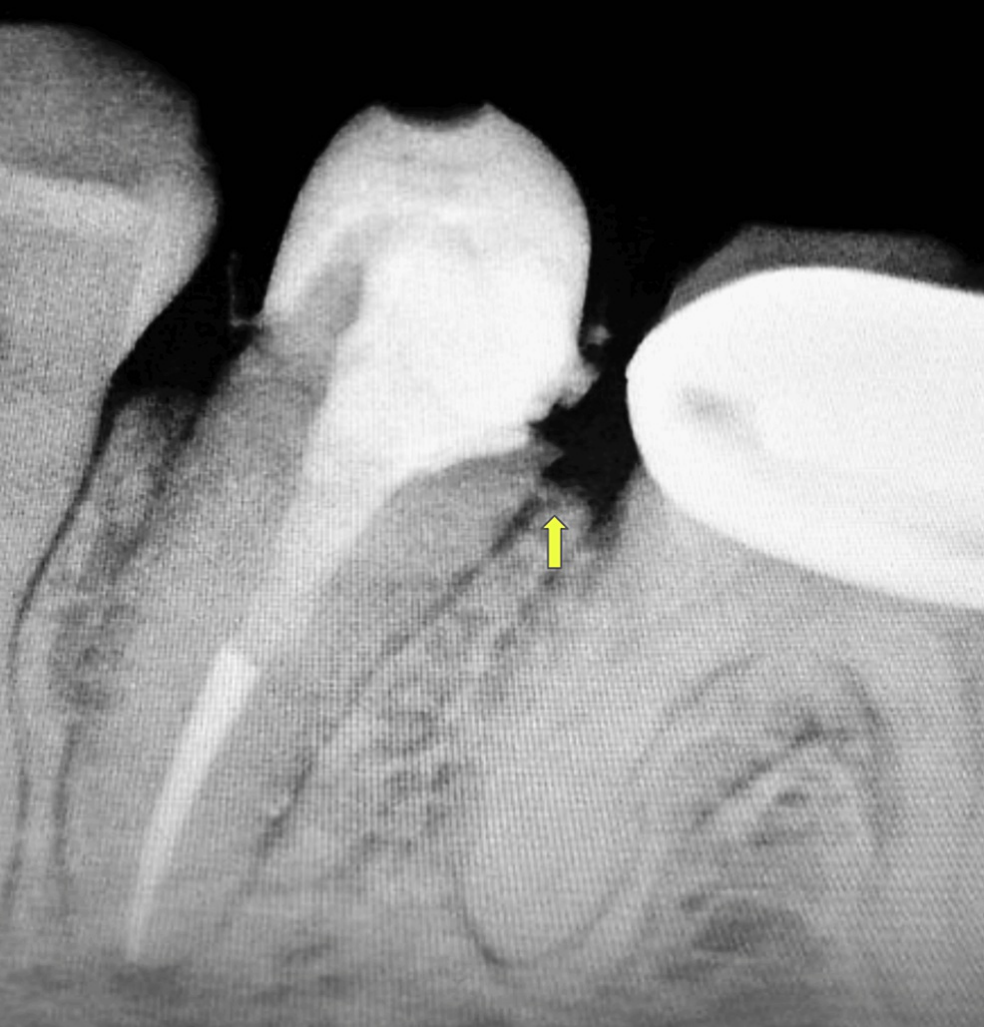
Intra-oral periapical radiograph showing crestal bone loss due to SCTA violation SCTA: Supracrestal tissue attachment

The patient had good oral hygiene and complied with the post-crown hygiene maintenance instructions. Under local anaesthesia bone sounding was done to evaluate the site. The measurement achieved was less than 2 mm from the prosthesis margin to the crest of the bone which was suggestive of SCTA violation. Radiograph revealed crestal bone loss suggestive of early periodontal disease with teeth 35 and 36 (Figure 2).

The treatment plan was to surgically access the site and recreate the SCTA, maintaining the positive architecture. The plan was to increase the length between the prosthetic margin and the bone level to re-establish the SCTA and avoid impingement of the crown margins. The surgical procedure included administration of 1:100000 (epinephrine) Lignocaine. An intra-sulcular incision using Bard-Parker handle with No. 15 scalpel blade (Aspen Surgical Products, Caledonia, MI, USA) was placed and a full-thickness envelope flap was raised involving the teeth (34, 35, and 36). The amount of flap raised was very conservative to access the required level of the alveolar bone. Periodontal measurements were repeated after the elevation of the access periodontal flap (Figure 1). Post evaluation the distance between the level of the crest of the alveolar bone to the prosthetic margin was planned to be increased so that the body could re-establish its SCTA. An end-cutting bur was used with a surgical handpiece to perform osteotomy under copious irrigation. Subsequent osteoplasty was done to recreate the positive architecture and re-confirmation was done using a periodontal probe. The periodontal flap was sutured using a silk suture (5-0). After surgery, the patient was evaluated for hemostasis. Post-operative instructions were given, and medication was advised. The medication prescribed was a pain killer (SOS). The patient was also advised to use regular 0.2% chlorhexidine mouthwash. The patient was followed up after one week for suture removal. The post-surgical period remained uneventful and the healing of the surgical site was satisfactory.

## Discussion

**Figure 3:**
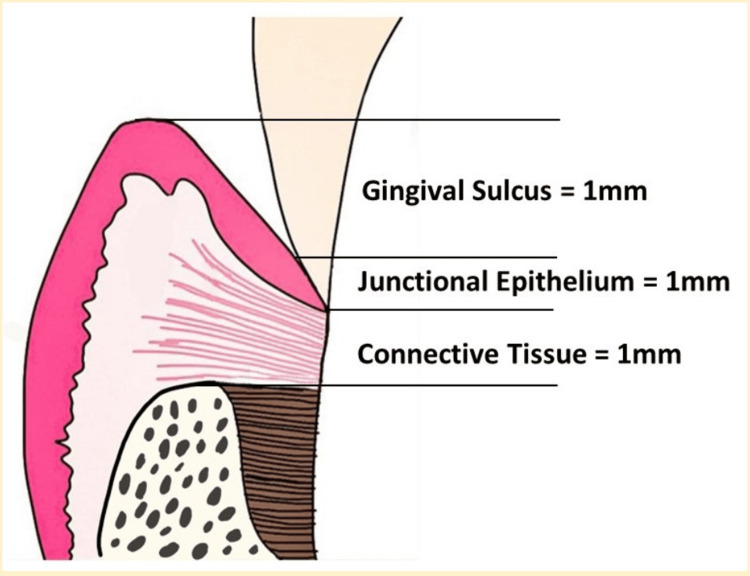
Figure representing average dimensions of SCTA SCTA: Supracrestal tissue attachment Image credit: Syed Wali Peeran, Tahir Bijli

Periodontal health depends on the preservation of SCTA, formerly known as biologic width. Each tooth's supracrestal connective tissue and junctional epithelium constitute this attachment (Figure 3).

**Figure 4:**
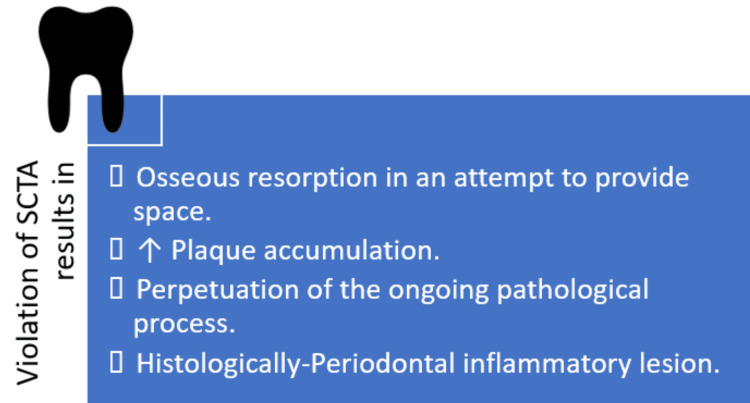
Consequence of violations of SCTA SCTA: Supracrestal tissue attachment Image credit: Syed Wali Peeran, Karthikeyan Ramalingam

Deeper placement of margins can lead to SCTA violation and can be detrimental to the health of periodontium, acting as a persistent irritant. Many studies have demonstrated adverse changes in subgingival microbiota, increased plaque index, progressive gingival recession, and deepened pocket depth with SCTA violations [8-12] (Figure 4).

Preserving the health of periodontium is very critical for the success of any restorative procedure. One of the main objectives of restorative prosthesis is to maintain gingival health which mandates the preservation of SCTA [12-15]. Table 1 shows the surgical approaches with gingivectomy and surgical crown lengthening procedures as a treatment strategy.

**Table 1:** Table showing surgical approaches as a treatment strategy

Treatment strategies			
Surgical approaches			
Gingivectomy	Surgical crown lengthening		
- 2 mm or more of gingival tissue remains after the procedure	Apical positioned flap without osteotomy	Apical positioned flap with osteotomy	Surgical extrusion
- Bone level is 2 mm or more apical to the cemento-enamel junction	- Inadequate amount of keratinized gingiva	- Adequate amount of keratinized gingiva	Intra-alveolar transplantation
	- Bone level is 2 mm or more from the cemento-enamel junction	- Bone level is less than 2 mm from the cemento-enamel junction	

The most commonly used surgical techniques are the apical shifting of the SCTA through respective techniques like surgical crown lengthening or the removal of excess gingival tissue employing gingivectomy to avoid violations [13]. The gingivectomy procedure is performed when there is an adequate amount of keratinized tissue and an SCTA width of greater than 3 mm on bone-sounding evaluation [14]. A 2 mm presence of keratinized tissue is estimated to be present post-gingivectomy. Hence, it is carried over in cases of altered passive eruption where the bone level is 2 mm or more apical to the cemento-enamel junction (CEJ) [15].

**Figure 5:**
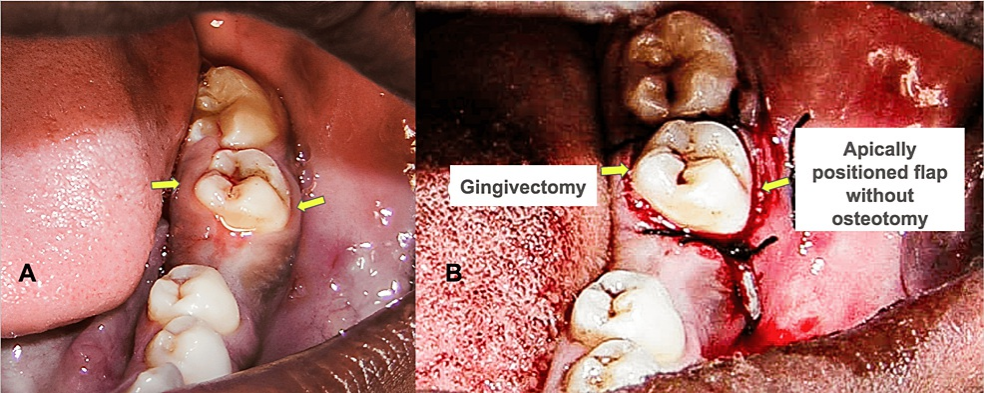
Clinical images A: Pre-operative image showing compromised crown height in teeth 37 B: Post-operative image of crown lengthening by gingivectomy on the lingual side and flap surgery on the buccal side Image credit: Syed Wali Peeran, Karthikeyan Ramalingam

Crown lengthening is indicated in cases with the placement of sub-gingival restorative margins. These restorations violate the SCTA, short clinical crowns, teeth with excessive occlusal/incisal wear, tooth fracture within the cervical third of the tooth, and in cases of unequal or unaesthetic gingival margin [9] (Figure 5).

In the case of single-rooted teeth, the tooth is repositioned into the socket in a more coronal or supragingival position, which is known as surgical extrusion [16-18]. Its advantages are that it can be done rapidly, leads to minimal bone loss, and better maintenance of the gingival papillary tissue. However, the lack of a universal protocol and the possibility of root resorption and ankylosis are the disadvantages of the procedure [17].

Table 2 shows the other approaches for the management of SCTA including the conservative and ultraconservative strategies.

**Table 2:** Table showing the ultraconservative and conservative approaches

Treatment Strategies					
Ultraconservative approaches	Conservative approaches				
Reattachment of tooth fragments	Orthodontic management				Deep margin elevation techniques
	- Slow orthodontic tooth eruption				
	- Forced rapid orthodontic tooth eruption with supracrestal fiberotomy				

In patients who are unable or unwilling to undergo periodontal surgery, Orthodontic tooth eruption(slow or forced ) is performed [13]. However, the forced orthodontic eruption of the tooth is contraindicated in cases with inadequate crown-root ratio or cases of lack of occlusal clearance for the required amount of eruption [19]. Deep margin elevation (DME) techniques have been proposed to for indirect restorations [20]. These are the available concepts for the management of SCTA width violations.

**Figure 6:**
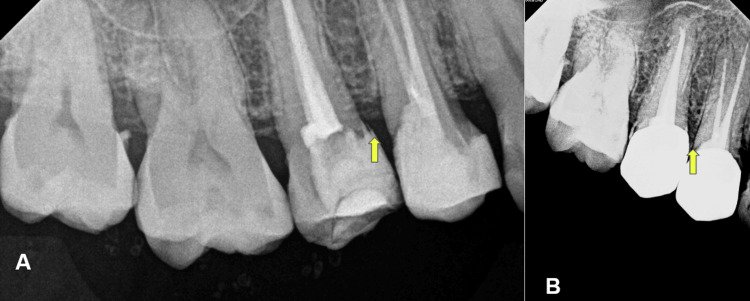
Radiographs showing deep margin elevation method performed on the mesial aspect of teeth 15 A: Pre-operative radiograph B: Post-operative radiograph Image credit: Syed Nahid Basheer, Reem Hassan Kelani

Proper tooth isolation and the use of composite resin are essential steps in these clinical procedures to avoid SCTA width violations [20]. Additionally, the R2-technique for deep occlusal-proximal resin composite restorations has been shown to prevent gingival and periodontal inflammation when the restoration margins do not violate the SCTA width [21] (Figure 6).

In cases where esthetic demands require margins to be hidden below the gingival margin, there is a risk of SCTA violation [22]. Therefore, an ultra-conservative approach, such as reattachment, should be careful of tooth fragments or use fiber posts when managing coronal tooth fractures to minimize biologic width violations [23]. Cone beam computerized tomography (CBCT) provides the most accurate and precise measurements of the bone height and thickness and Cementoenamel junction position [24,25]. CBCT also enables determining distances between the CEJ and the facial bone crest, CEJ, and gingival margin, and CEJ and alveolar bone crest [26]. It is also helpful in designing precision custom-made surgical guides for crown lengthening surgery [27].

Vacek et al. concluded that the average SCTA was 2 mm, similar to an earlier study done by Gargiulo et al. reported a similar average of 2 mm [28,29]. However, the study by Gargiulo et al. also showed a range of SCTA from 0.75 mm to 4.3 mm. This indicates that the SCTA was patient-specific, which suggests that a specific SCTA assessment must be performed for each patient [28]. Our case report shows the need for regular, mandatory SCTA evaluation before placement of the restorative margin and the need for the restorative margin not to violate SCTA to maintain gingival health and the success of the prosthesis. 

## Conclusions

In restorative dentistry, supracrestal tissue attachment (SCTA) violation is a serious issue since it can harm the periodontal health and the longevity of restorations. The main treatment advised to prevent violation and produce the required supracrestal tooth length is surgical crown lengthening. Clinicians can promote optimal periodontal health and restoration lifetime while managing instances involving biological width violation by employing conservative procedures such as fragment reattachment and adhering to proper protocols.
